# Permanent Scaffolding of Social Expertise

**DOI:** 10.1007/s10441-026-09533-8

**Published:** 2026-06-25

**Authors:** Anya Plutynski, Ge Fang

**Affiliations:** 1 Department of Philosophy, Washington University in St. Louis, St. Louis, MO 63130 USA; 2 Data Science Institute, The University of Chicago, Chicago, IL 60637 USA

**Keywords:** Scaffolding, Expertise acquisition, Social learning, Jazz improvisation

## Abstract

The metaphor of “scaffolding” originates from developmental psychology, and has become a central theoretical concept that plays explanatory roles in psychology, developmental biology, and evolutionary biology. In developmental psychology, the scaffolding process is typically characterized as a child’s temporary reliance on external structure in training activities, which leads to long-term transformation of the child’s capacities. In recent attempts to clarify this concept for wider usage (e.g., Caporael, Griesemer, and Wimsatt 2014a, b; Neto et al. 2023), the temporariness of dependence on scaffolds is still seen as a typical, if not defining, feature of scaffolding explanations. In this paper, we follow Bill Wimsatt’s footsteps (Wimsatt 2014a, 2019; Wimsatt and Griesemer 2007) in examining the development of skills that require complex developmental trajectories. Our cases are jazz improvisation and scientific expertise, both of which are distinctively social and improvisational. We argue that the social scaffolds required to reach the maturity of such expertise are continually required thereafter for maintenance and further development. This is because improvisational training is somewhat indistinguishable from improvisational performance, and performance in social expertise aims at interactions, communications, and rapport between interlocutors.

## Introduction

All of the contributors to this volume – whether their philosophical interests, skill, arguments, or methods – have directly or indirectly been scaffolded by Bill Wimsatt’s teaching, writing, or thinking. But, what exactly is “scaffolding”? How are we scaffolded in our learning, or in the development of expertise? What role does interpersonal exchange play in scaffolding expert performance? Is social scaffolding essential to expertise, or more significant in some domains of expertise, as distinct from other domains?

The notion of “scaffolding” has played an important role in explanations in psychology, developmental biology, and evolutionary biology. “Scaffolding” is a central theoretical concept in these fields, almost as pervasive as “information,” “genetic code,” or “mechanism.” Unfortunately, when such concepts come to play such a central role, there is a danger of their becoming overly diffuse in their application. Some worry that the notion of “scaffolding” has become so expansive as to refer to any and all environmental conditions to some system that facilitates the production of an outcome. This would seem to permit too much. We would not want to say, for instance, that the mere presence of oxygen is a scaffold for skilled tool use.

Recently, Neto et al. (2023) offer a general account of scaffolding explanations, in service of specifying more precise conditions on such explanations. They describe eight notable features present in scaffolding explanations: (1) they are contrastive; (2) the scaffolding makes an outcome of interest more likely; (3) the scaffold is “independent of or external to the system”; (4) “the system actively responds to the presence of the scaffold, using it or adjusting to it to produce the outcome of interest”; (5) “the scaffolding process causally sustains the activity of the system”; (6) it is temporary; (7) “it typically transforms the system, providing new skills, properties, or capacities that it did not have before”; and thus, (8) it opens up a new space of possibilities, not available previously. (Neto, et. al., 2023, p. 7–8).

We find much to recommend this analysis. Our account builds on Neto et al.’s account by providing a more detailed exposition of key causal factors at work in steps (5) and (7). We draw upon empirical work on expertise, attending to the ways in which expertise is optimized in different domains, specifically, jazz and science.

Where we depart from Neto et al. is with respect to item (6) above. Our central thesis is that community scaffolding is essential to continued expression and optimization of expertise in domains high in sociality and improvisational skill. We are thus expanding upon the notion of scaffolding as typically understood, so as to allow scaffolds to be “permanent.”^1^ Psychologists have long emphasized how social learning is essential to the development of certain forms of expertise, but they have typically illustrated this via appeal to central episodes of learning. We extend this model of episodic scaffolding, and characterize the distinctive ways in which certain kinds of expertise require extended process of maintenance and continued development over time. In service of this end, we distinguish scaffolding episodes and scaffolding “trajectories,” and describe how they relate structurally, where the former are temporary features of the latter. A “scaffolded trajectory” contains a specific order of scaffolding episodes modulated partially by a learner’s ability to construct and maintain social relations, and partially by various kinds of group structures, such as individual-teacher relations, small friend groups, social institutions, and nationwide associations.

Drawing upon Fang (2025), we argue that different forms of optimal training in expertise acquisition target different forms of “excellence”. Fang makes a distinction between three “dimensions” of excellence: virtuosity, strength, and improvisation. Domains high on the dimension of “virtuosity,” for instance, exhibit excellence in executing complex motor movements over a short period of time (high in classical piano and violin, or gymnastics). “Strength” is excellence in resisting obstacles (typical in domains such as cycling and swimming); “improvisation” is excellence in responding to fluctuating circumstances in real time (high in domains such as jazz improvisation). To these three dimensions, we add “sociality”: excellence in forming and utilizing social connections (high in domains such as science). Each domain of expertise has a specific combination of dimensions upon which expertise is more or less dependent. Examples of domains high in sociality and improvisational skill are jazz performance, orchestra performance, science, team sports, public speaking, and psychotherapy. In this paper, we will use two case studies to illustrate our central thesis that domains of expertise that require permanent social scaffolding are those high in sociality and improvisational skill: expertise in jazz musicianship and expertise in the sciences.

The social scaffolds we mentioned above are essential for social and improvisational expertise to persist and continue to develop. The reason for such permanent dependance is twofold. First, unlike many purely virtuosic domains, improvisational training cannot be easily distinguished from improvisational performances. Second, social scaffolding is constitutive of optimal performance in domains where expert activity is collaborative. Unlike solitary hunting, forms of expertise that either require collaboration in producing meaningful results or which aim at mutual understanding, effective communication, and rapport-building, require interpersonal exchange and dynamic interaction with others (Sterelny 2003, 2010). For these collaborative enterprises, social scaffolds are not temporary.

The organization of the paper, and arc of the argument for our thesis, is as follows. In Sect. 2, we offer a historical overview of the origins of the concept of scaffolding in psychology. In Sect. 3, we articulate the structure of scaffolding episodes and scaffolding trajectories. In Sect. 4, we consider the first of two examples of domains of expertise in which sociality and improvisational skill are high: Jazz. In Sect. 5, we consider the case of science. In Sect.  6, we conclude 2.

## Historical Origins

The original context in which “scaffolding” appears in the scientific literature is psychology, specifically, Vygotsky’s (1978) account of human learning. Vygotsky argued that humans are distinctive in their capacity to use language and other symbolic communication. For these symbols to be “internalized,” humans rely *essentially* on perspective taking – i.e., representing to oneself how others perceive or think in response to one’s actions.

By “internalization,” Vygotsky (1978) had in mind the “internal reconstruction of an external operation” (p. 56), a process by which children were capable of representing plans for guiding their own action. He noticed that when children are challenged to solve practical tasks, they often narrate the solution “out loud,” or use what he called “egocentric” speech, e.g., “On the stool, I could reach the candy with the stick…” Narrating out loud, in his view, was a transitional state between social or collaborative learning and autonomous planning or problem solving, part of the process of “internalization” of social speech. Vygotsky offers another vivid example of internalization: the process by which very young children learn how to point at objects. This example is a vivid illustration of the essential role social interaction plays in learning, according to Vygotsky, an illustration we will return to below in our discussion of social scaffolding:

Initially the gesture is nothing more than an usuccessful attempt to grasp something… the child attempts to grasp an object beyond his reach; his hands, stretched toward that object, remain poised in the air…. When the mother comes to the child’s aid and realizes his movement indicates something, the situation changes. Pointing becomes a gesture for others. The child’s unsuccessful attempt engenders a reaction … *from another person*. Consequently, the primary meaning of that unsuccessful grasping movement is established by others. Only later, when the child can link his unsuccessful grasping movement to the objective situation as a whole, does he begin to understand this movement as pointing… it becomes a movement aimed at another person….” (p. 56).

When a child learns to point, he is internally representing to himself another’s understanding of his desires; that is, he is coming to recognize that his mental state can be recognized by others, and communicated symbolically. What begins as a merely reactive behavior (reaching for something one desires) is transformed via interpersonal exchange into a cognitive representation of symbolic communication, an essential part of child development and language learning. Vygotsky describes this as another instance of the process of “internalization”: “Every function in the child’s cultural development appears twice: first on the social level, and later, on the individual level; first, between people (interpsychological), and then inside the child (intrapsychological)… All the higher functions originate as actual relations between human individuals.” (Vygotsky 1978, pp. 56–7).

Vygotsky’s model of the “zone of proximal development” grew in part out of this picture of children’s learning. The zone of proximal development is the “distance between the actual development level as determined by independent problem solving and the level of potential development as determined through problem solving under adult guidance or in collaboration with more capable peers.” (Vygotsky 1978, p. 87). The “zone” represents how “independent” a learner is of these external scaffolds required for learning. For better and for worse, this picture of learning, by which one moves from interdependence to autonomous problem-solving, became tremendously influential in the psychology literature. Particularly as Vygotsky spoke of this capacity to “internalize” as the “qualitative leap from animal to human psychology”(p. 57), many took away from his view the notion that reliance on external scaffolds is an immature state, and freedom from scaffolds as “mature.” Thus, many psychologists assumed that an essential feature of scaffolding is that it is temporary.^3^

Yet, a close read of Vygotsky shows that he does not as a matter of fact think *all* forms of learning *require throwing away the social scaffold*. He writes: “For many many functions, the stage of external signs lasts forever, that is, it is their final stage of development. Other functions develop further and gradually become inner functions.” (p. 57) Our project here in part aims to build on this idea. We will argue that in some domains, persistent scaffolds are key elements of sustained, optimal expression of expertise.

Among the first uses of “scaffolding” in the psychological literature was that by Wood, Bruner, and Ross (1976), who explain how their view is distinct from others as follows:

Discussions of problem solving or skill acquisition are usually premised on the assumption that the learner is alone and unassisted. If the social context is taken into account, it is *usually treated as an instance of modelling and imitation*. But the intervention of a tutor may involve much more than this. More often than not, *it involves a kind of "scaffolding" process that enables a child or novice to solve a problem, carry out a task or achieve a goal which would be beyond his unassisted efforts. This scaffolding consists essentially of the adult "controlling" those elements of the task that are initially beyond the learner’s capacity, thus permitting him to concentrate upon and complete only those elements that are within his range of competence*. (Wood, et. al., p. 90)

Wood et al. above contrast their account with one where learners simply imitate or model their behavior on the tutor. In their view, social learning is not simply a matter of imitation. Rather, the tutor has to attend carefully to the learner’s current capacity (or, “zone of proximal development”) and “scaffold” the child’s learning by designing or adjusting the task in such a way that the learner can focus on the elements that they are currently capable of addressing.

By way of example, they discuss a particular task they designed in which a tutor instructs children between the ages of 3–5 to build a pyramid-shaped block structure, using blocks with pegs that fit into one another. Tutors were instructed to enlist interest, “let the child pace the task for himself as far as possible,” and only offer instruction at particular stages, in a manner carefully attuned to the child’s level of skill and capacity to receive and make use of help. “Scaffolding” involved adjusting instruction as needed to enable a child to make progress. Wood et al.’s model of a “scaffolded” process breaks down as follows:

1. **Recruitment**. … enlist the problem solver’s interest in and adherence to the requirements of the task.
2. **Reduction in degrees of freedom**. … simplifying the task… regulating feedback.
3. **Direction maintenance**. Learners lag and regress to other aims… The tutor has the role of keeping them in pursuit of a particular objective.
4. **Marking critical features**. A tutor … accentuates certain features of the task that are relevant.
5. **Frustration control.** “Problem solving should be less dangerous or stressful with a tutor than without”.
6. **Demonstration.** Demonstrating or "modelling" solutions to a task, when closely observed, *involves considerably more than simply performing in the presence of the tutee*. … the tutor is "imitating" in idealized form an attempted solution tried (or assumed to be tried) by the tutee in the expectation that the learner will then "imitate" it back in a more appropriate form. (Wood, et. al., p. 98)

Wood et. al.’s work here is significant in its careful attention to the dynamic interaction between teacher and learner, or how instruction has to be “individualized”. “Scaffolded” instruction, on this view, involves inter-personal adjustment of instruction, with close attunement to the learner, their level of interest, stage of development, and level of frustration. Without the instructor’s capacity to generate a “model” of the learner’s “performance characteristics,” the child will become frustrated, and will not become an independent problem solver. Effective instruction, on this view, requires that the instructor see the world through the child’s eyes, and help them *learn how* to *solve future problems on their own*. We will return to this point, below, when we consider distinctively collaborative forms of expertise.

A similar view is defended by Bickhard (2005), who refers to the capacity to learn how to learn as a kind of “self-scaffolding.”^4^ Like Wood, et. al.’s model, learning on Bickhard’s view is “recursive,” in that it is sensitive to, and dependent on prior learning. On Bickhard’s view, environmental factors (or features of the problem itself) can block a problem-solving path. Selectively eliminating (or, “blocking”) such factors permits individuals to find simple, intermediate solutions to problems, that can then be used to build further skills. Key to his view is that this process can be carried out without the assistance of others; for instance, individuals might choose “simple cases to work on first, by moving to idealizations, by breaking down into subproblems, by making use of resources that are currently available but may not always be available, and so on” (Bickhard (2005), p. 170). For Bickhard, individuals can adopt some of the very same strategies that are typically associated with “external” scaffolds, i.e., teachers: “blocking” barriers to problem solving, breaking down tasks, selectively working on one at a time, idealizing one’s model of the task, and giving oneself reminders.

Key to Bickhard’s view is that “Functional scaffolding enables models of ongoing self-scaffolding …” and “it makes sense of the notion of permanent scaffolds, which may make possible various interactions and task accomplishment that simply would not be possible otherwise, and for which the scaffolds need to be permanently available… social organization and language have properties of such permanent scaffolding.” (Bickhard (2005), p. 171) What is striking about this picture for our purposes are first, that Bickhard defines “scaffold” functionally, not structurally: it is what the scaffold *does* that defines the scaffolding, not whether self-directed or “externally” guided, temporary or permanent. Second, for Bickhard, the continued or ongoing reliance on social organization or language allows for the possiblity of thinking of scaffolds as permanent, rather than temporary, and for the social “architecture” of continued expertise development as part of this permanent structure.

What seems to have happened in this literature is that the notion of a “scaffold” has become more articulated and sophisticated, such that merely episodically being of assistance in improving the production of some outcome at some specific stage of learning is not enough for understanding how humans learn, and continue to learn. Rather, “optimal” scaffolds (at least in some contexts) help the learner to learn how to solve problems, and this may involve coming to rely upon and create external scaffolding (including social scaffolding) in service of continued development of expertise. We refer to this as the building of a scaffolding trajectory, or a network of scaffolding episodes.

There seem to be several key elements of scaffolded models of learning from the psychological literature, which, as we will see, yield several open questions about the scope and import of “scaffolding” as a conceptual construct in modeling expertise:

1. All seem to agree that scaffolding is a process of learning, often (though not always) involving “external” forms of support, where these may take various forms: social interaction, overt instruction, or “prostheses” of various sorts (reminders to self, etc.). Not all models of scaffolding require that the external support be a person or involve interpersonal interaction, though this is typically emphasized, particularly in early phases of development.
2. It has often been assumed that an essential feature of scaffolding is that a scaffold must eventually be discarded or set to one side. However, even Vygotsky seemed to grant that some “scaffolding” is permanent.
3. It’s often assumed that the goal of learning is independence or autonomous problem-solving. However, as theories of how learning is scaffolded were articulated, a key element of seeking out ways to scaffold one’s continued learning seemed to be relianceon social networks.
4. Scaffolding has come to be associated with a rejection of passive models of social learning, where learning occurs via rote memorization or imitation. A key element of scaffolding (to count as such) by the lights of the above theorists is that it requires a dynamic interaction between learner and tutor, with individualized attunement to the individual learner’s stage of development, and adjustment to instruction on an individualized basis.

Where does this leave us? It seems that there are several open questions in the literature on scaffolded learning, arising in part out of the historical evolution of this concept, which involved adapting it to different contexts. Among such questions are the central organizing ones in this paper: How exactly is expertise optimized? Do different kinds of expertise require different types of scaffolding? How ought we to think about the relationships between temporary (or episodic) scaffolds and lasting scaffolds? What features are distinctive of the latter? Which lasting scaffolds (or ongoing scaffolding trajectories) are particularly relevant to some forms of expertise? Are there some forms of expertise, such that, for expertise to be sustained, “self-scaffolding” requires collaboration?

In our view, the best characterization of the process of scaffolding (and thus what should count as a scaffold) may well depend on the kind of skill developed, or domain of expertise. While there may be many differences in the details, broadly speaking, all scaffolded learning involves some form of selective attention. How that selective attention is directed (whether by external factors, internal, or both) might depend importantly on which context of learning we are concerned with: the infant learning how to point, the child of 3–5 building blocks, building specific (virtuostic) skills, such as music performance, or becoming a scientist. In some contexts, the problems of selective attention are solved by external scaffolds, and in others, by “self-scaffolds”; in some by both. In some contexts, the goal is elimination of external social scaffolds; in others, building and maintaining a robust social scaffold is key to optimization of expertise.

## Expertise, Scaffolding Trajectories, and Structural Scaffolds

In this section, we distinguish between scaffolding “episodes” and scaffolding “trajectories.” A “scaffolding episode” is a process in which a change comes about in some system or entity via reliance on some external or internal structure. Exemplary scaffolding episodes include viruses relying on a host cell to replicate, a student using flashcards to aid memory, or an injured person using a cane to rehabilitate. In contrast, a scaffolding trajectory is an extended process of psychological development, which includes many scaffolding episodes. We choose the term “trajectory” to capture, *in part*, the network of conditional relations among different scaffolding episodes, in which later scaffolding episodes are conditioned on the result of earlier scaffolding episodes. Students’ progression towards expertise is often planned, controlled, or modulated either by an instructor, a group, an institution, and of course, by the students themselves.

This distinction between scaffolding episodes and trajectories has several motivations. First, it helps clarify whether, and in what sense, a scaffold is temporary. We acknowledge that many scaffolds are temporary. However, in domains where social skills and improvisational skills are essential to expert performance, social scaffolds are permanent; indeed, in some cases, interaction with the scaffold becomes a central aim of expert development and performance.

**Fig. 1:**
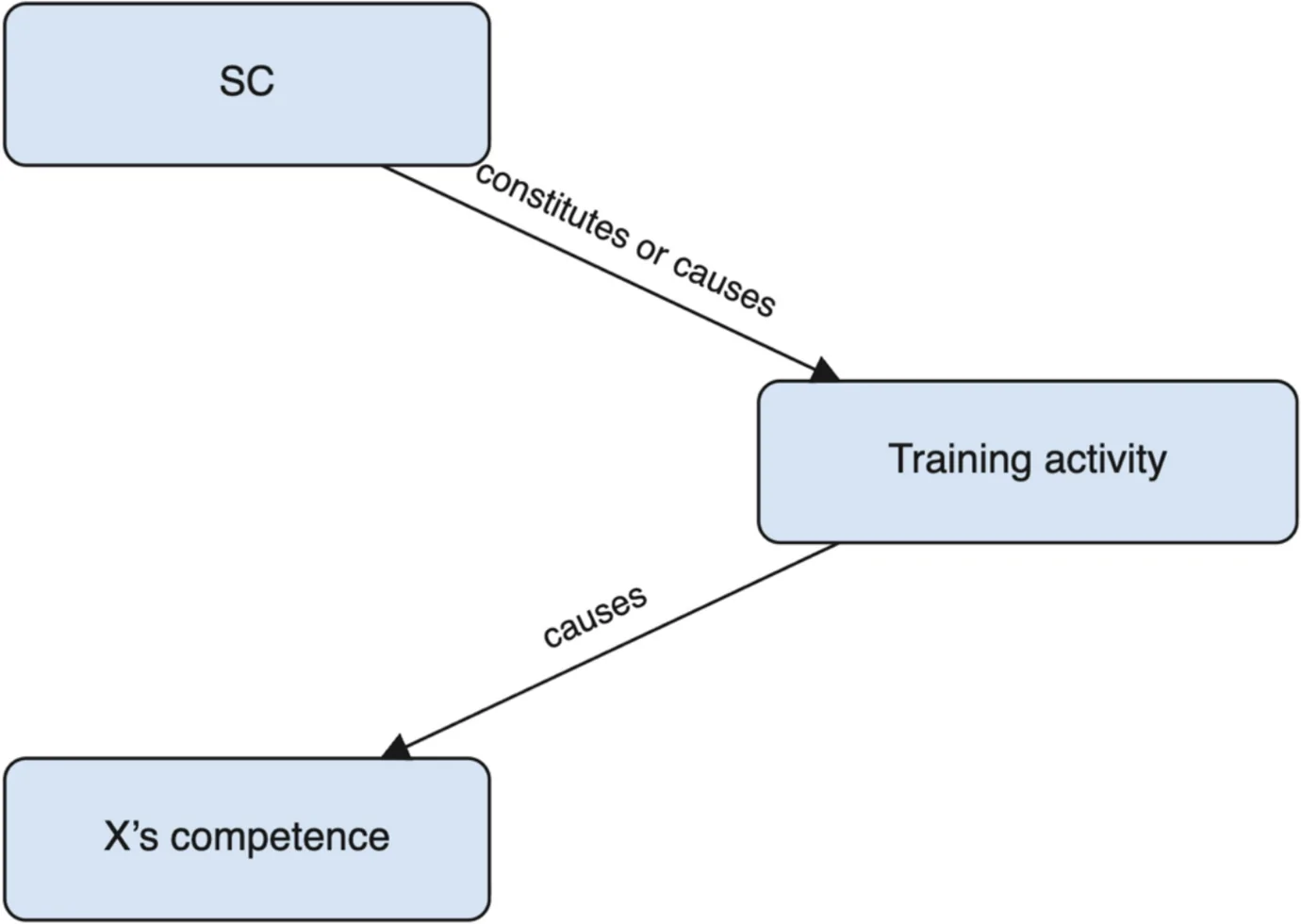
A schematic representation of a scaffolding episode

A scaffolding episode is a *pair* of relations. Using SC to refer to the scaffolds and X to the system under development, the episode captures the relations between SC and activity, and between activity and X’s specific competence. In short, SC either causes or constitutes^5^ a training activity that leads to the development of the competence. See Fig. 1 for a schematic representation of a scaffolding episode.

To illustrate, consider Vygotsky’s characterization of the development of pointing behavior. Initially, the mother’s reaction to the child’s “grasp” (her realization that the child indicates something) constitutes the symbolic meaning of the child’s “pointing” behavior, without which the behavior is but an unsuccessful attempt at grasping. The mother’s symbolic interpretation is a “scaffold” (i.e., the SC) for the child’s observation of the correlations between the grasp-like bodily movement and its social effects on others. This case exemplifies the relation between SC and the training activity. Recurrent “point” behavior, with the help of the mother, leads to the transformation of the child’s competence, via the “internalization” of the symbolic meaning of the gesture of “pointing.” The scaffolding episode starts with the onset of the recurring behavior and ends with the internalization of the symbolic understanding.^6^ The events described above correspond to Neto et al. (2023)‘s criteria (5)—“the scaffolding process causally sustains the activity of the system”—and (7)—“it typically transforms the system, providing new skills, properties, or capacities that it did not have before”.

**Fig. 2:**
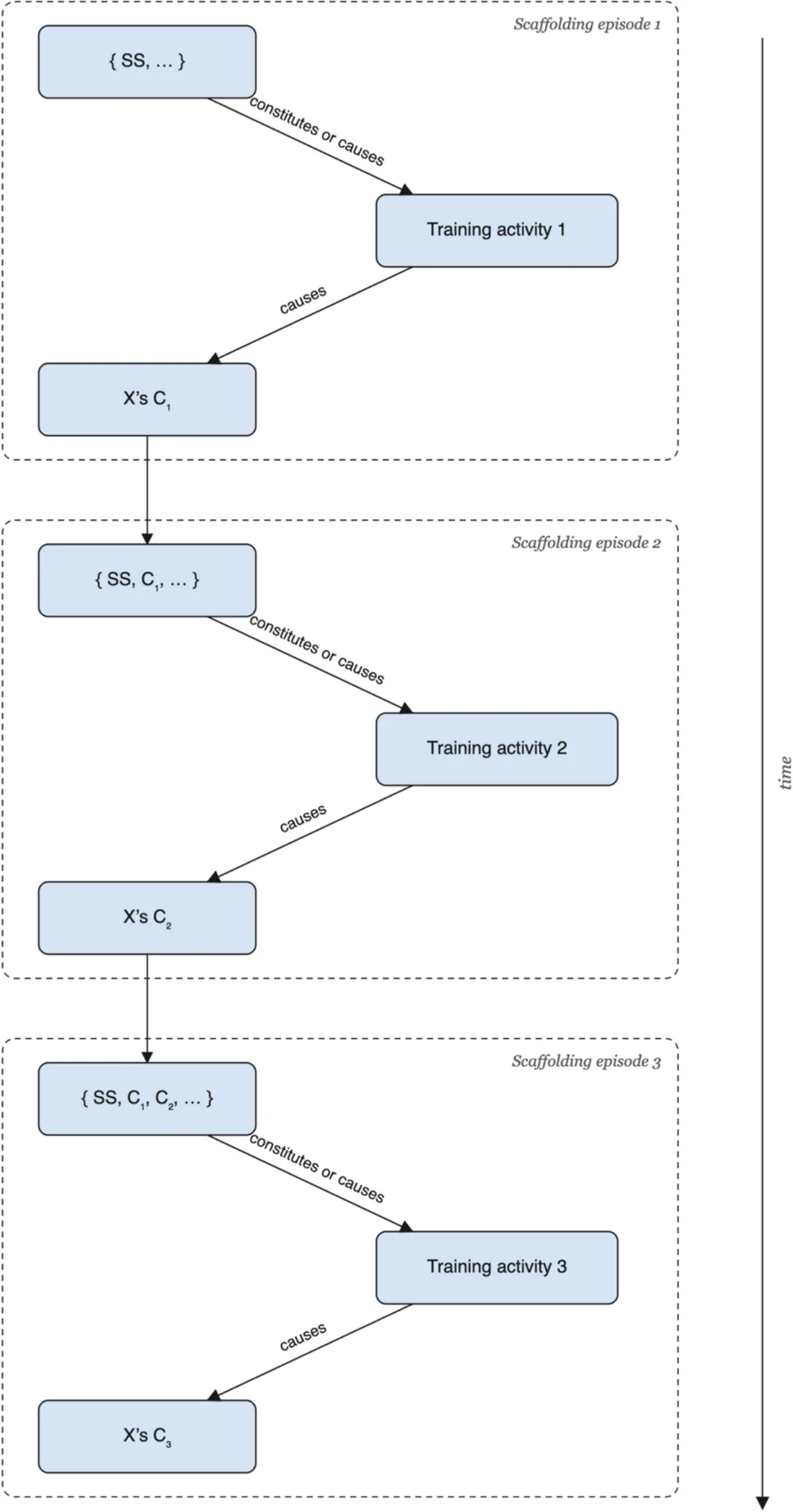
A scaffolding chain linking several scaffolding episodes. The actual development process will contain many more episodes and does not necessarily look like a chain (e.g., see Fig. 3)

The developmental processes for complex expertise involve more heterogeneous phases and processes. We define a s*caffolding trajectory* as a network of scaffolding episodes connected by conditional relations, in which a shared set of educational resources both (1) serve as the episodic scaffolds for most, if not all, scaffolding episodes in the network, and (2) plan, monitor, control, and maintain the student’s progression in the network. In the context of expertise acquisition, the scaffolding trajectory starts at the first lesson and ends at a conventionally determined point of expertise maturity. We call this shared set of educational resources *structural scaffolds* (SS in Fig. 2)*.* The structural scaffolds may include educators, institutions, communities, routine practices, or self-development plans. See Fig. 2 for a schematic depiction of a simple scaffolding trajectory:

A scaffolding trajectory fits Neto et al. (2023)‘s criteria (5) and (7). The causal interactions between the structural scaffold and the agent sustain the activity of the agent and transform the agent. The effects of scaffolding consist not only in first-order activities, i.e., causal or constitutive contributions of educators or institutions, but also their second-order role in planning, monitoring, controlling, modulating, and maintaining the progression of development. As the examples in Sect.  2 show, educators design tasks suitable for students after understanding what students can and cannot do—this is a social, dynamic interaction.

The notion of “scaffolding” that was invented and refined by early developmental psychologists refers, at least in part, to scaffolding trajectories. By focusing on conditional relations, this notion is also more closely connected to Wimsatt’s notion of generative entrenchment. When Wimsatt (2014a, b) says that "[e]ntrenched features commonly act as scaffolding," he refers to scaffolding trajectories rather than episodes. Not all SCs in scaffolding episodes are entrenched parts. Only some structural scaffolds, as well as the learning processes they engender at the start of a scaffolding trajectory, are entrenched. These early stages in scaffolding trajectories “play a larger generative role”: They shape the conditions that play scaffolding roles for later scaffolding episodes. The content and manner of interaction between teachers and novices may be fixed in some domains of expertise—particularly domains where virtuosity is high, and deliberate practice is essential to learning. Deliberate practice, as originally formulated to characterize optimal training in classical music, involves forming ideal images of music in the mind and repeating a short phrase while aiming to replicate those images (Ericsson et al. 1993).^7^

Jazz is one domain with such entrenched, structural scaffolds. The basic “vocabulary” of jazz, i.e., the typical licks, phrase fragments, and references to famous passages, resembles basic units in standardized combinatorial systems. Wimsatt (2014a, b) introduces units in standardized combinatorial systems as a typical example of an entrenched part of cultural evolution, with examples such as standardized sets of nuts and bolts, standardized alphabets, or standardized interfaces of software and hardware in electrical devices. In jazz, the entrenched part of the practice includes not only combinatorial units but also “Real Book” compositions, their chord progressions, and techniques for wielding musical instruments. All of these occupy the basic layers of the conditional network.

By distinguishing and describing scaffolding episodes and scaffolding trajectories, this section sets up the conceptual framework for presenting our thesis that social scaffolds are essential for the scaffolding trajectories of social and improvisational domains of expertise. These domains do not have a clear distinction between training activities and performing activities, and social scaffolds often constitute their performing activities. Jazz musicianship is not merely an example for illustrating the conceptual framework, but also a case study to illustrate this point, which we will turn to in the next section.

## The Case Study of the Development of Jazz Improvisation

In this section, we dissect the development of jazz improvisation as a concrete instance of a domain of expertise high in both sociality and improvisational skills. Jazz is particularly well-suited to showing how the social and improvisational dimensions interact with the other dimensions of expertise development, and that excellent social improvisation generally occurs late in conditional networks.

Throughout our characterization of jazz, we will appeal to the jazz-language analogy, which is frequently drawn by jazz musicians and scholars. According to them, the proper auditory perception and performance of jazz etudes, discrete patterns, phrase fragments, blue licks, bebop licks, signature licks (e.g., Charlie Parker licks, Sonny Rollins licks, Charlie Rouse licks), shout patterns, swing figures, as well as references to past jazz, popular, or classical pieces, are like the acquisition of vocabulary. Improvisational capacities, or the capacities to organize these elements into coherent jazz phrases in real time, are like creating new sentences under a set of grammatical rules. Personalization is comparable to speaking with one’s own literary style and content. David Sudnow’s *Ways of the Hand,* a report of decades of jazz piano learning, heavily utilizes the jazz-language parallel (Sudnow 2001). Before grasping any jazz vocabularies, Sudnow describes playing on the piano as skin to saying “noinoinoinoinoinoinoinoinoinoino” (p. 20). Efficient, beginner-level vocabulary training relies on rote repetitions such as saying “the book, the book, the book book book the book, the book, the book book book” (p. 39). Performing a jazz phrase is to combine jazz vocabulary in a way that has “a sentence structure” (p. 57), when students strive to connect “short phrases”, “sentence fragments” into “adult jazz utterances” (p.120).

We will start by delineating the scaffolding trajectory and the structural scaffolds of jazz improvisation before turning to the reasons why these scaffolds are essential for expertise maintenance.

### The Scaffolding Trajectory

The different scaffolding episodes of jazz development recounted in Sudnow (2001)—the grasping of word pronunciation, the accumulation of vocabulary, and the patterned organization of that vocabulary into sentences—are nodes in conditional networks of the developmental trajectory. This subsection elaborates on the conditional relations among these episodes, the complexity of the conditional network, and the heterogeneity of developmental modalities across scaffolding episodes.

Sudnow defines a preliminary stage of vocabulary building as “knowing where to go”, and characterizes it as fundamental: “if you don’t know where you’re going you can’t go anywhere correctly" (p. 20). Sudnow compares his disastrous attempt to improvise in a jam session without adequate vocabulary training to Charlie Chaplin on the assembly line in Modern Times. Improvisation requires a prior grasp of some basic vocabulary, partly because they function as a fail-safe for improvisation.

The conditional link between vocabulary training and improvisational training does not capture the full developmental trajectory in jazz. Beyond fluency lies innovation in the strongest sense: contributions that transform the tradition, opening new possibilities that other musicians then explore and adopt. Dizzy Gillespie’s harmonic innovations, for instance, “made people aware of the flatted fifth and the flatted ninth—which actually goes back to Bach fugues,” as George Duvivier recalls (Berliner 1994, p. 168). Relatively few musicians achieve this level of innovation, where individual experimentation becomes a communal resource.

A great deal of familiarity with jazz is already required before vocabulary acquisition can start. Novices must first build familiarity with jazz standards and their underlying harmonic progressions. Lonnie Hillyer discovered the necessity of harmonic knowledge when he sat in with Miles Davis’s band as a teenager and lost his place after eight bars; Davis pulled him off stage with the gruff assessment: “You don’t know your chords, do you?” (p. 71).

**Fig. 3:**
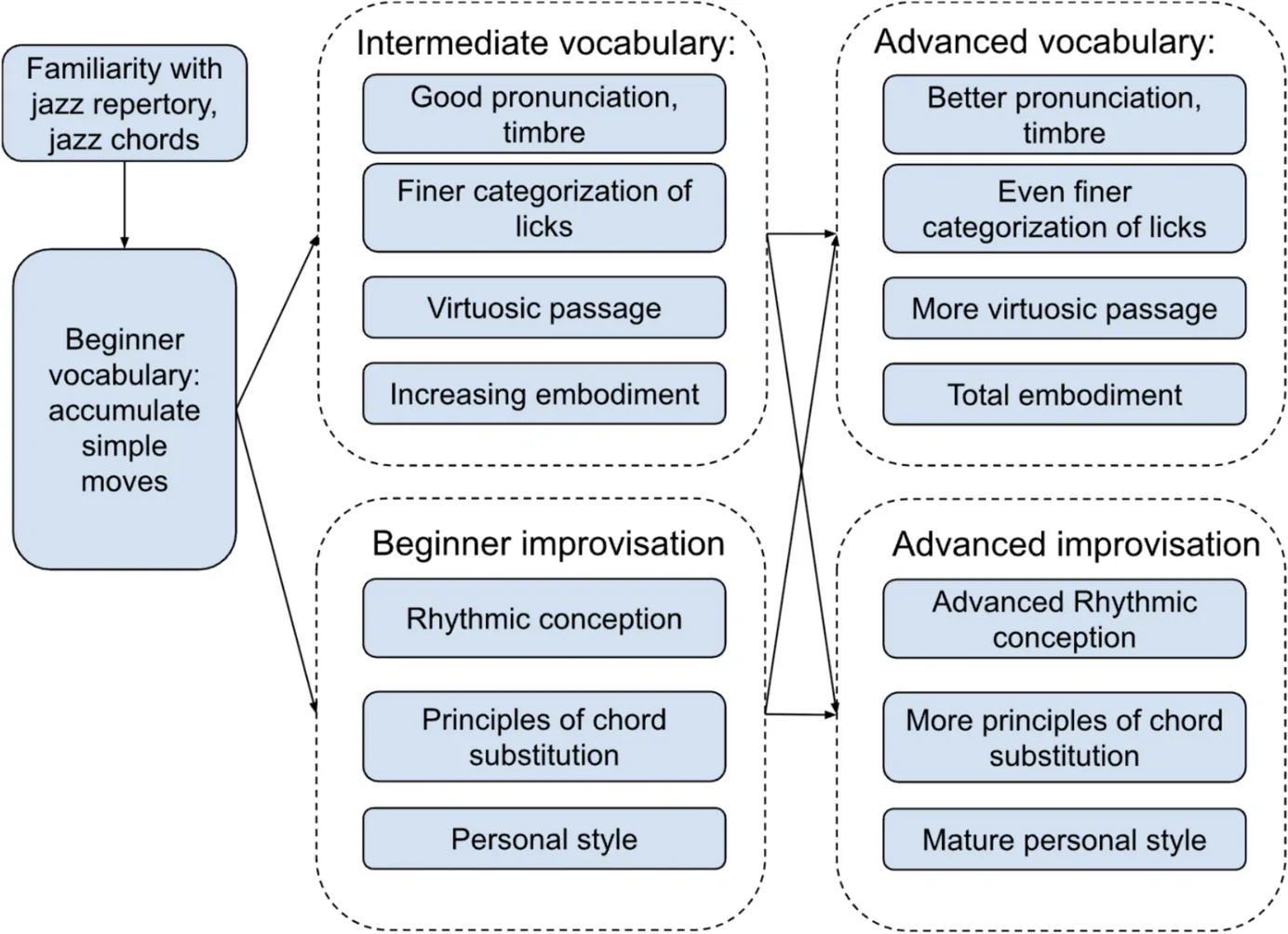
A partial conditional network in the development of sub-skills in jazz improvisation

These phases or sub-phases in the development of jazz improvisation are distinct scaffolding episodes. They have slightly different goals and methods. Both vocabulary training and improvisational training consist of multiple sub-dimensions, with progressions across these dimensions following complex interdependent relations (see Fig. 3 or a rough illustration). Efficient methods for training music vocabulary, for instance, differ from those for developing improvisational abilities. The beginning stages of “vocabulary-building” may benefit from mindless repetitions. The later stages of vocabulary training, in which the musician attempts to develop virtuosic passages, fine-grained recognition of variations, optimal efficiency of motor execution, and the exact control of timbre and evenness, often require deliberate practice.^8^ Neither mindless repetition nor deliberate practice is optimal for improvisational training. To improve improvisation, it is often more efficient to imitate more experienced players, vary the contexts, or simply practice it in contexts similar to actual performance.

The temporal initiation, transitions, and distribution of these heterogeneous training episodes are partially controlled by the structural scaffolds of the trajectory, to which we now turn.

Structural scaffolds are developmental resources that guide, monitor, or control the initiation of heterogeneous developmental phases and play various causal or constitutive roles across multiple phases. The structural scaffolds for jazz development involve various social structures, as well as individuals’ capacity to establish and maintain them. Such structures include jazz teacher-student relations (2 people), task groups (~ 5 people), demes (~ 30 people), and macrodemes (~ 300 people). This division follows Caporael’s classification of *core configurations*, which are “subgroups of face-to-face groups posited to repeatedly occur throughout human evolutionary history as a joint function of subgroup size and modal tasks.” (Caporael 2014, p. 61; also see Caporael 1997). From forager studies, Caporael abstracted the “magic numbers” 2, 5, 30, and 300, corresponding to the caregiver-infant dyad, hunting task groups, band, and macroband.

In jazz, as in most academic fields, teachers have an important influence on their students that shapes various phases of technical, improvisational, and social development. Consider Barry Harris, a jazz pianist famous for his teaching method. Harris’s teaching method is scaffolded by years of interacting with his students (Berliner 1994, p. 47), which, in turn, prepares students at various stages of their development. In the early phases of vocabulary building, Harris’s sessions transformed into seminars where students learned to discuss and analyze music theoretically (p. 38); in later stages of sentence-level improvisation and personalization, Harris pushed students toward independence. Students also form relations with many teachers and collaborators. One of Barry’s students explains that “Barry and his contemporaries prepared us for guys like Mingus” (p. 47), and “working with Charles Mingus on and off for those twelve years” was “like school” (p. 49). His work with his teacher prepared him to collaborate with other “teachers,” who also became collaborators. In this way, performing is a kind of extended apprenticeship.

The developmental influence of Jazz bands, friend groups, or cohorts is similarly comprehensive. Red Rodney describes Charlie Parker’s band as “graduate school,” and George Duvivier describes Coleman Hawkins’s band as “the University” (p. 49). These groups offer immediate corrective feedback, which accelerates the deliberate practice required for ingraining musical vocabulary. When Tommy Flanagan and his teenage peers gathered to learn Charlie Parker tunes from records, “one guy would try to play a tune from a new Bird record, and someone else would say, ‘No, that’s not right,’ and we’d hash it out together” (Berliner 1994, p. 64). As learners progress to combining vocabulary into short phrases and sentences, small groups provide low-stakes opportunities for real-time improvisation with supportive interlocutors. For instance, Curtis Fuller describes daily sessions with John Coltrane where “Coltrane would say, ‘Hey Curtis, try to play this on the trombone,’ and I would try to run something down. I’d struggle with it and he’d say, ‘You’re getting it’” (p. 39). The patience and encouragement possible only in intimate settings allow learners to work through the “stammerings and stutterings” characteristic of this phase without the evaluative pressure of larger audiences. Beyond technical scaffolding, small groups provide crucial emotional support that sustains motivation through the decade-long developmental trajectory. For instance, Jackie McLean reports that Bud Powell was “always dropping by the house and playing with him, encouraging him to develop, and inviting him along on gigs” (p. 38).

Larger organizations, such as societies, school programs, and regular jam session attendees, provide additional structural scaffolding. The Bebop Society in Indianapolis and the New Music Society in Detroit “promoted more formally organized sessions” stratified by ability: the New Music Society included both high-caliber groups and less advanced groups of younsters (Berliner 1994, pp. 42–43). Broader jazz culture, city-wide networks, or national associations could be analogous to macrodemes. The jazz community functions as a distributed educational system and a hub for information exchange. Such venues provided what smaller groups could not: exposure to the full diversity of the tradition. Iconic venues like Small’s Paradise Club, or what Tommy Turrentine dubbed “Paradise University”, brought together four generations of musicians where newcomers could witness masters like Parker, Gillespie, and Bud Powell (Berliner 1994, pp. 43–44). Opportunities to interact with musicians from a different city, join a different band, or participate in different jam sessions can provide further guidance and motivation for remedying flawed learning strategies. For instance, a beginner may mistakenly believe that they can get away without learning how to transpose standards into new keys. John McNeil only realized this after joining a jam session in which the saxophonist “called the tunes in different keys.” He went into a panic, “hid from other musicians for months” until he relearned his repertory “in all twelve keys” (Berliner 1994, p.66).

The jazz community also served as a hub for sharing idiosyncratic learning techniques adapted to available technology. Musicians discovered that “early record players had controls enabling listeners to slow a record’s speed by gradations until they could catch a particularly fast passage, albeit at a lower pitch, transposing the retarded phrase into its original pitch immediately thereafter. Those lacking such equipment slowed the turntable by applying slight finger pressure to the record” (Berliner 1994, p. 96). This technique not only made practicing rapid passages accessible but inadvertently trained students to transpose phrases across keys.

The mere presence of social scaffolding in the development of jazz improvisation does not distinguish jazz from domains that are non-social or non-improvisational. What is distinctive about jazz, as well as other domains require high sociality and improvisational skills, is that the social scaffolds are essential to the continued exercise of expertise after its maturity. Next, we explain why the improvisational and social characteristics of jazz improvisation that render these social scaffolds indispensable.

### Learning by Doing

Quite generally, expertise deteriorates if not maintained. The research on expertise maintenance is continuous with those on expertise development, because the training activities for expertise maintenance and development are quite similar. For non-improvisational skills, development/maintenance is not necessarily performance, but can be dedicated practice. This is why social scaffolding, once it allows the practitioner to grasp how to practice deliberately, does not need to persist for further development and maintenance. Improvisation training, however, is a form of learning by doing. Maintaining improvisational expertise requires continuous performance. Without opportunities to perform, a jazz musician can maintain their virtuosic skills through tailored exercise, but not their improvisational skills. The performance in jazz improvisation, in particular, aims to socialize, so social scaffolding is required. This subsection will discuss the blending of development activities and performance in jazz, and the next subsection will discuss the social character of jazz performance.

To learn improvisation, one must start improvising, as it cannot be dissected into smaller parts. One always learns by doing. Other improvisational domains in general also favor this approach (Fang 2025, chapter 3). In domains ranging from foreign language acquisition to software engineering and philosophy, the actual conversation, problem-solving, paper-writing are essential parts for improving improvisational skills.

The aim of “learning by doing” differs from that of technical preparation or theoretical understanding. The aim of the former is to better understand dynamic contexts. The latter aim to abstract away specific contexts and subtleties in the service of focused exercises with good skill transfer. However, when understanding context is essential to an activity, no amount of technical preparation can fully prepare students for it. Sudnow’s early experience of a jam session vividly demonstrates that all his technical preparation could not substitute for the actual experience of improvising in real time (Sudnow 2001, pp 32–25). Despite having practiced “nearly all day," the pathways he had learned could not save him from the chaos of real improvisation.

One effective method for improvisational training in jazz, as in programming, linguistic, and sporting contexts, is thus exploration across varying contexts. To some extent, students can simulate such variation in isolated, controlled environments. For instance, jazz students are encouraged to play the same chord progression in different keys, fill the same progression with varying notes of a chord, vary the spacing between notes, use different voicings (emphasizing notes within a chord), and change the rhythms. Exploratory practice in the context of music development is like linguistic experimentation, where different words and phrases are employed in different contexts to probe their flexible uses.

However, simulations of contextual variation cannot be a sufficient replacement for the contextual fluctuations in actual improvisation. The dynamic moments in actual performance can only be experienced as such. Design needs keep changing. So is the mood of bandmates, the attention of the audience, and the status of the debate in philosophy. The value of apprenticeship, often seen in expertise development, is that it offers a gateway to experience the actual contexts of engineering, music performance, and academic debate.

So far, we have use jazz to illustrate that an improvisational domain requires actual performance for expertise maintenance. Formally, this claim does not yet entail that jazz requires social scaffolding for expertise maintenance, since the concept of improvisational performance does not imply the need of social scaffolding. Solo-hunting, for instance, is an improvisational domain, in which the contextual variation arises from unique environments and intelligent animal behavior. A socially scaffolded mature hunter can maintain their expertise by hunting alone. In contrast, the dynamic, unique contexts in jazz improvisational are due to the social, interactive nature of the domains. In such social domains, social structures that allow for interaction with other improvisers are indispensable for performance activities.

### Socialization as a Purpose

Jazz improvisation is a type of conversation. Berliner (1994) titles a chapter of his ethnography “The Collective Conversation,” and notes that musicians liken “group improvisation to a conversation that players carry on among themselves in the language of jazz” (p. 348). This framing is not merely descriptive but normative. Communication failures produce poor performances. As Chuck Israels observes, “If the relationship between the bassist and the drummer is not working, you know that right away. It’s just painful if we can’t agree” (p. 396). Jazz musicians who only hear themselves when they play miss what makes jazz work (p. 400).

Musicians constantly listen to and respond to one another in real time, creating chains of call-and-response. A strategy in which this is both practiced and used in performance is “trading fours.” This is a jazz convention, where musicians alternate short improvised phrases, responding to “the most general features of each other’s phrases” to create “such continuity between the parts that the resultant line sounds as if conceived by one mind” (p. 369). Even the rhythm section’s foundation requires what Berliner calls “striking a groove”, or a “shared sense of the beat” that emerges through mutual adjustment (p. 349; also see Monson 1996 for an ethnography on the interactiveness of jazz’s rhythm section). As Kenny Barron summarizes: “It’s a matter of give and take” (p. 348).

When interaction succeeds, musicians describe profound experiences of connection that transcend mere coordination. Franklin Gordon captures this: “Everyone’s locked in there together… These are the magical moments, the best moments in jazz” (p. 388). Ronald Shannon Jackson articulates the deeper significance: “This music is really about the relationships between all the players. When the relationship is happening, you don’t hear piano, bass, and drums…. You hear the total communication of individuals” (p. 389). Melba Liston describes how “everybody can feel what each other is thinking…. You breathe together, you swell together, you just do everything together, and a different aura comes over the room” (p. 392). In this way, social skill – in particular, the capacity to mentalize, or imagine what it is like to live inside another’s mind – is a key dimension, along which expertise in jazz performance is optimized. Keith Copeland speaks of anticipating a soloist’s ideas “almost telepathically,” so that “we end up playing phrases together that match each other. It’s like we’re talking together at the same time” (pp. 389–390). These descriptions suggest that social connection is not merely instrumental to good jazz but also partly constitutive of what musicians aim at.

Even apparently solitary improvisation is covertly social. Max Roach explains that “when I play, it’s like having a conversation with myself,” with each phrase responding to what came before (p. 192). Lonnie Hillyer extends this, describing how he thinks of himself “as being two players” when improvising call-and-response patterns (p. 195). More tellingly, Hillyer notes that once musicians absorb conventions for sympathetic interaction, “they soon begin to imagine responses of rhythm section players to their own improvisations, even when practicing alone” (p. 359). Solitary performance requires mentally reconstructing the social context that makes improvisation meaningful.

While becoming more familiar with the “language” and interactive contexts of jazz performance, jazz students also build their own style and voice. This process requires careful study of existing voices. Walter Bishop Jr. describes it as a type of “assimilation” that welds together bits and pieces from different musicians into something personally distinctive (Berliner 1994, p. 120). Barry Harris and his peers, after having idolized and listened to “all the giants”, feel the need to “see out a bit,” gradually discovering which elements resonate with their own sensibilities (Berliner 1994, p. 121). Tommy Turrentine would take “anything that pleased [him], anything that sounded beautiful. It might be a phrase. It might be a scale. It might be anything.” (Berliner 1994, p. 139). Through such selective appropriation during improvisational practice, musicians develop preferences about which patterns, rhythmic approaches, and timbral qualities to favor. Lonnie Hillyer observed that “everyone who studied [under Barry’s system] came out sounding different” (Berliner 1994, p. 167). Originality emerges not from ignorance of tradition but from comprehensive familiarity with explored musical spaces combined with authentic self-understanding—a never-ending process in which a musician, like a philosopher, may display markedly different musical personalities at successive stages of development.

To conclude our case study of jazz, the communicative nature of jazz ensures that the structural, social scaffolding in the trajectory of a maturing jazz musician is required in jazz performance, which in turn is required for the maintenance or further development of the matured expertise. Like Vygotsky’s suggestion that language is a permanent scaffold, individuals’ sociality, teacher-student relations, jazz bands, and jazz communities are permanent scaffolds for jazz improvisation. They are always part of the performance and thus part of the ongoing development of jazz improvisation.

## Comparison and Contrast with Scientific Expertise

Barbara Wimsatt (2014b) has drawn upon the notion of scaffolded cognition to give a constructive account of the emergence and persistence of expertise—using examples from two prominent scientists (Craig Ventor and Charles Darwin). Wimsatt draws in part for this on the work of two sociologists of science, Collins and Evans (2007), who have defended the view that scientific expertise is distinctive, in that it is essentially social and interactive. Our view of scaffolding episodes as nested within scaffolded trajectories builds upon both the above accounts.

Collins and Evans (2007) draw attention to the distinctively social character of science by distinguishing between two forms of expertise, “contributory” and “interactional” expertise. The former “is what we normally mean when we talk of experts—these are people who exercise their expertise by contributing to their specialist domain.” (Collins 2018, p. 72) In contrast, “interactionist” expertise is a kind of skilled ability to engage with one’s community, a capacity that depends on “possession of the tacit knowledge of the specialist domain.” This is a skill that takes some effort to build and establish – and, it requires what we have called a socially “scaffolded” trajectory – the continued development of a community of practitioners with whom we communicate and share knowledge, language, practices, skills, and so on. Collins writes, “To become an expert in some domain is a matter of becoming embedded in the social life of the domain, acquiring what is to a large extent, tacit knowledge, so as to internalise the associated concepts and skilful actions to the point of fluency” (Collins 2018, p. 68).

In other words, the development of “interactionist” expertise of the sort to which Collins and Evans refer consists in the development of not only individual knowledge, and skills, but also the right kind of social network – an extended, scaffolded, social trajectory. As Longino (1990), and Solomon (2007) (and many others) have argued, scientific practice essentially involves collaboration, and critique. In this way, it is inherently “social,” requiring social learning not only in service of individual scientists’ development of skillful technique, vocabulary, etc., but also in service of the collaborative work that leads to and enables the ongoing growth of knowledge in their field of specialization. In our language, the “sociality” of a domain is a matter of degree, dependent on how social skills are key to developing and maintaining expertise, and thus is also a reflection of how indispensable social embeddedness is to one’s continued expert performance. Drawing upon research into the sociology of expertise acquisition in science, or what Collins and Evans called the “Studies of Expertise and Experience (SEE)”, they generated what they called the ‘Periodic Table of Expertises’, where “level of expertise grows with embedding in the society of domain experts; the key is the transmission of domain-specific tacit knowledge…Under SEE, domains can be big or small so there can be ‘ubiquitous tacit knowledge’, such as natural-language-speaking or other elements of general social behaviour…” (Collins 2013, p. 253) Scientific communities are an exemplary instance of communities that depend upon ubiquitous tacit knowledge; in its uses of language, experiment, implicit assumptions about the appropriate range of problems worth solving, and exemplars of good solutions. The Periodic Table of Expertises (Fig. 1 in Collins 2013) characterizes a process by which experts pass into the stage of fluency, or “specialist expertise,” via acquisition of “‘Dispositions’, which describes certain individual abilities …” “‘Meta-expertises’” or “expertises we use to judge other experts” and “‘meta-credentials’. Collins and Evans are pointing to a variety of scaffolds that together promote and allow for the persistence and continued development of scientific expertise. Scientists must first learn “how to learn,” i.e., have the patience, facility, and social skills necessary to gain “tacit” knowledge of the language, concepts, experimental methods and techniques required for mastery and contribution to their field of expertise. Moreover, skilled learning is continuously fostered by the scientific community, via giving and receiving critical feedback, sharing data, techniques, etc. Scientists work collaboratively, building upon one another’s research, and one can be better or worse at collaborative work – this is what Collins (2004) calls “interactional” expertise. In several ways, this process of transition from “fluency” toward “meta-expertise” echoes parallel processes described above in the progress of jazz musician’s skill set—where a musician first begins to acquire fluency in the mastery of the “language” of jazz—or, acquires skill at musicianship, and then becomes more skilled at engaging with other musicians. Of course, the kind of skills in question differ, but their method of acquisition, and its dependence on social networks, is similar. Collins writes:

The underlying idea of the Periodic Table is that the acquisition of nearly every expertise, if not all of them, depends on the acquisition of the tacit knowledge pertaining to the expert domain in question. Tacit knowledge can be acquired only by immersion in the society of those who already possess it. Therefore, the process of moving to the right-hand end of the Specialist Expertise line depends on becoming socially embedded in the appropriate groups of experts so that one can acquire ‘specialist tacit knowledge’ … The process is social though the outcome is real—an ability to do and understand things that one could not do and understand before… The two right-hand categories of the Specialist Expertise line indicate that there are two kinds of socialisation that can lead to two kinds of specialist expertise. The rightmost category—contributory expertise—is what is normally thought of as an expertise, and it is the practical expertise that enables one to contribute to a domain of practice. To acquire contributory expertise one must work within the expert domain. Interactional expertise, on the other hand, can be acquired by deep immersion in the linguistic discourse of the domain alone. (Collins 2013, p. 254).

This distinction – between “contributory” and “interactional” expertise – bears a striking similarity to the distinction between skilled musicianship, or virtuosity (the sense of skill typically attributed to expert musicians), and the kind of skill at social, improvisational engagement typical of jazz musicians. Likewise, in science, this process consists in moving from mastery of knowledge, a language (theoretical terms, etc.) to mastery of a “form of life,” or set of experimental and research practices, which require social engagement. Collins uses the example of scientific expertise to argue for a social “Cartesianism,” emphasizing his departure from the “Cartesian” notion of knowledge as being a property exclusively of individual knowers: “The special thing about humans is their ability to feast on the cultural blood of the collectivity.” (Collins 2010, pp. 125–131) In science, this “blood” is the various components of a research program, its collective products, and activities: the language, experimental practice, theory, problem space, probable solutions, etc. In other words, scientists build upon, and rest their expertise development on, the collective enterprise of science overall, which is a social enterprise, and requires collective action.

Similarly to Colliins and Evans, Wimsatt (2014b) uses two case studies of famous scientists as exemplary cases of how expertise is scaffolded. In her words:

Cognitive development is intertwined with career development within the process of professionalization. I adopt a social or *situated* perspective on cognitive development within this framework and bring together the concepts of scaffolding, entrenchment, and generativity as explanatory. I also see this as happening through dynamic coordination and integration of various resources over time and with a focus on the interactions between mentors and trainees. (Wimsatt 2014b, p. 342).

Wimsatt relies upon Vygotsky (1978), Tomasello (1999), Wimsatt (1986, 2001), and Sterelny (2012) to develop her account of social cognition and its role in the socialization and expertise development of scientists. All view cognition as socially mediated, and building incrementally over time, where sociality (and social learning, or mentorship) is essential to preservation and transmission of knowledge, and development of skilled problem-solving. According to Wimsatt, scientists, their cognitive skills and abilities, and the systems or social networks of which they are a part, collectively enable or support a generative process for further development of both scientific expertise and the scientific enterprise itself. Both individuals and groups are in ongoing interaction. These interactions typically involve problem-solving. And, problem-solving trajectories are “stabilized through some or many bases or foundations that can be built upon (which I call footholds) and scaffolded through various kinds of assistance or supports (which I refer to as handholds).”(p. 345).

She illustrates these concepts of “footholds” and “handholds” with brief narrative reconstructions of the scientific careers of Craig Ventor and Charles Darwin—both of whom relied both on their didactic training in science and medicine, and on the social support and facilitation of their interests by mentors and colleagues, not only in their training, but over the course of their careers. Craig Ventor went from being a mediocre student and Vietnam veteran to the inventor of PCR. His experience as a medic in Vietnam, and his learning the laboratory technique for isolating cells at UCSD were what she refers to as “footholds” along the way to his eventual scientific career. His mentorship by distinguished scientist Nathan Kaplan was a “handhold” that enabled him to explore his interest in cell receptors. A foothold may be a “crucial decision, specific book, lecture, or mastery of some significant knowledge that was inspirational for setting someone on a particular path.” (p. 346) In contrast, “handholds are assists or scaffolds in the process of traversing a specific developmental trajectory.” Crucial to Wimsatt’s account of Craig Ventor’s scientific career is the social network he relied upon to gain access to the scientific community’s resources, and to promote his novel technique, in service of a new research program: namely, the sequencing of the first human genome.

In Darwin’s case, Wimsatt argues, his footholds included an early interest in naturalism and beetle collecting, whereas “handholds” included his befriending Henslow, whose mentorship and support led to Darwin’s voyage on the Beagle, meeting Sedgewick, and reading Lyell on uniformitarianism. In all these ways, Henslow furthered Darwin’s interests, and perhaps as importantly, his career in science. It was Henslow who read Darwin’s field notes at the Cambridge Philosophical Society, and promoted their publication. Wimsatt grants that the distinction between footholds and handholds can sometimes be blurred. Nonetheless, in her view, footholds and handholds are a part of an ongoing developing cognitive system, or “problem-solving trajectory.” Such a trajectory may be viewed as continuous with, and integrating, the research laboratory, or community of scientists, who together pose and solve problems, develop novel solutions, and collectively and individually enable the furthering of research. We see this view as providing a yet further example of the importance of social scaffolding in not only enabling scientists to “gain” expertise (at the individual level), but also to promote and maintain expertise, as part of the social trajectory.

Social scaffolds are required to sustain and promote “interactionist expertise,” which refers to the variety of ways in which scientific expertise depends on skilled social interactions. There are several such skills and interactions that are essential to continued maintenance and development of expertise, as illustrated by the examples above: using the critical feedback of other scientists, collaborating in research, whether in early stages of formation of viable hypotheses, experimental design, construction of technologies for data gathering, or development of methods of assessment and measurement. For instance, Darwin’s *Origin* was, arguably not simply the product of his mind and observations alone, but depended essentially on the expertise of his vast community of correspondents and teachers. When writing and researching the *Origin*, Darwin was in constant contact with plant and animal breeders, as well as plant and animal collectors around the globe, all of whom allowed him to arrive at his laws of inheritance, illustrations of selective breeding, and observations of biogeographic distribution (Browne 1996). Without this correspondence with Asa Gray, for instance, he would not have been able to anticipate and respond to creationist objections to his theory, and admit where work yet needed to be done. He relied on his correspondence with geologists to arrive at theories about the vast age of the earth, and many transformations in the earth’s surface, as well as patterns in the geological strata.

In sum, collaboration and criticism are essential to the continued maintenance and development of scientific expertise. Scientific activity depends essentially on reciprocity and interaction, particularly given the manner in which scientific disciplines are continuously innovating from new data, changing experimental technique, technological development, transformations in data representation and analysis, theoretical advances, to new strategies of measurement as well as statistical tools. It is only in this interactive activity that expertise can be both maintained, and furthered. Barbara Wimsatt’s discussion of the “footholds” and “handholds” of both Ventor and Darwin illuminates how patterns of engagement among scientists is ongoing.

There are a variety of similarities and differences between jazz and science. First, we see jazz performance and science as both distinctively social. Excellent performers are experts not only in their virtuosity or musicianship, but in their capacity to listen to, respond, and engage with others in improvisation. Improvisational skill and social skill are thus importantly intertwined in jazz. A poor improviser is not only poor at trying out new “licks,” but at listening to and communicating with others. In some ways – depending of course on the discipline – scientific research is also essentially social. Scientists form communities – whether correspondents, working groups, laboratories, scientific associations, or societies. These communities are essential to the production, assessment, and communication of scientific knowledge. So, “sociality” is high in this domain, in that scientists need to be good at communicating with one another, receiving feedback, identifying points of disagreement, resolving conflict, and working together to common purpose. In some ways, this is akin to expert performance in jazz music, insofar as scientists need to learn to adopt a habit of listening carefully to others, and adapting to new information, technology, and so on. Second, both scientists and musicians learn by doing. Third, group size – as considered by Caporael – does seem to play an important role in the process of training future scientists, and so also maps neatly between science and jazz. A major difference is that the kind of skilled virtuosity distinctive of musicianship is quite different from the kind of knowledge-based expertise typical of science. Though, if Collins and Evans are correct, there is also a good deal of “tacit” skill involved in science as well, whether in the sense of physically embodied, or not formally articulated.

## Conclusion

One central aim of this paper is to elaborate on the proper meaning of scaffolding in the context of expertise development. In service of this end, we distinguish the scaffolding trajectory from scaffolding episodes. Whereas a scaffolding episode is a process in which a system transforms with the help of an external or internal structure, a scaffolding trajectory is an extended, potentially enduring process that consists of distinct scaffolding episodes connected through a network of conditional relations. As we illustrated with the case of jazz, various social structures, such as teacher-student relations, peer groups, jazz institutions, and national associations, not only provide resources for building specific skills but also control, monitor, and initiate transitions between scaffolding episodes. These social structures scaffold the developmental trajectories of expertise. The notion of scaffolding trajectory, as well as the observation of the second-order functional roles of the relevant social structure, can apply to most expertise with prolonged training processes and a complicated curriculum.

Drawing upon this important distinction, we then argue for two central theses: First, that some domains of expertise are high in the dimensions of sociality and improvisation. Second, that the persistence of social scaffolds – an instance of structural scaffolds in scaffolding trajectories – are key to both maintenance and further development of experts in these domains. We chose two vivid examples of cases as evidence: Jazz improvisation and science, though we take there to be many other potential instances of expertise that depends on persistent social scaffolding. In these cases, domain-relevant development is often not clearly distinguishable from domain-relevant performance. Jazz musicians and scientists learn by doing. Because of their social nature, social scaffolds that structure the developmental trajectory are continuously required to maintain and optimize expertise. In this sense, scaffolds are not always temporary.

One might wonder whether there anything more to say at the general level about the features of certain domains where scaffolds might be expected to be permanent. We take it that there are a range of potential views on this question, which it would take future work to develop. On the one hand, one could argue that there are some distinctive features of such domains, for instance, where successful social interaction is itself key to maintenance of expertise, perhaps because the products of such enterprises are distinctively collective efforts (an improvisational jazz performance, a scientific research tradition). Given that successful social interactions in such domains requires distinctive capacities, which can atrophy without practice, perhaps continued interaction is required to maintain and promote expertise. Depending on the domain, the social skills involved might be effective communication skills (e.g., listening skills, to ease or facility with language), emotional regulation, self- and other-awareness or “mindedness” (sometimes called “mentalization”), bodily awareness, or dyadic synchrony. All of the above capacities are reinforced via continuing social interactions, and tend to atrophy otherwise. And so, highly social domains are ones where interactions promoting such skills has to be continuously engaged. On the other hand, one might argue that the development of all expertise requires the capacity to search out and participate in the co-construction of new scaffolds, and to find collaborative partners for feedback and reinforcement. So these social skills and their practice are not distinctive or uniquely important to some domains of expertise. So, rather than take a stand on this bold (and important) question here, we leave this question open for future work.

Socially entrenched scaffolds have both advantages and disadvantages (Wimsatt and Griesemer 2007). There is always a danger of prior modes of practice preventing the development of innovations and problem-solving techniques. One limitation of our analysis is that we do not explore both the costs and benefits of these socially maintained scaffolds, nor how different types of social structure may vary in their roles in modulating the trajectory of expertise development. For instance, stringent structures of learning or apprenticeship may prevent innovation. Detailing how the function of social structures in development and performance vary across different phases of expertise development and post-development, both enhancing and constraining improvisational skill requires further analysis.

**Fig. 4:**
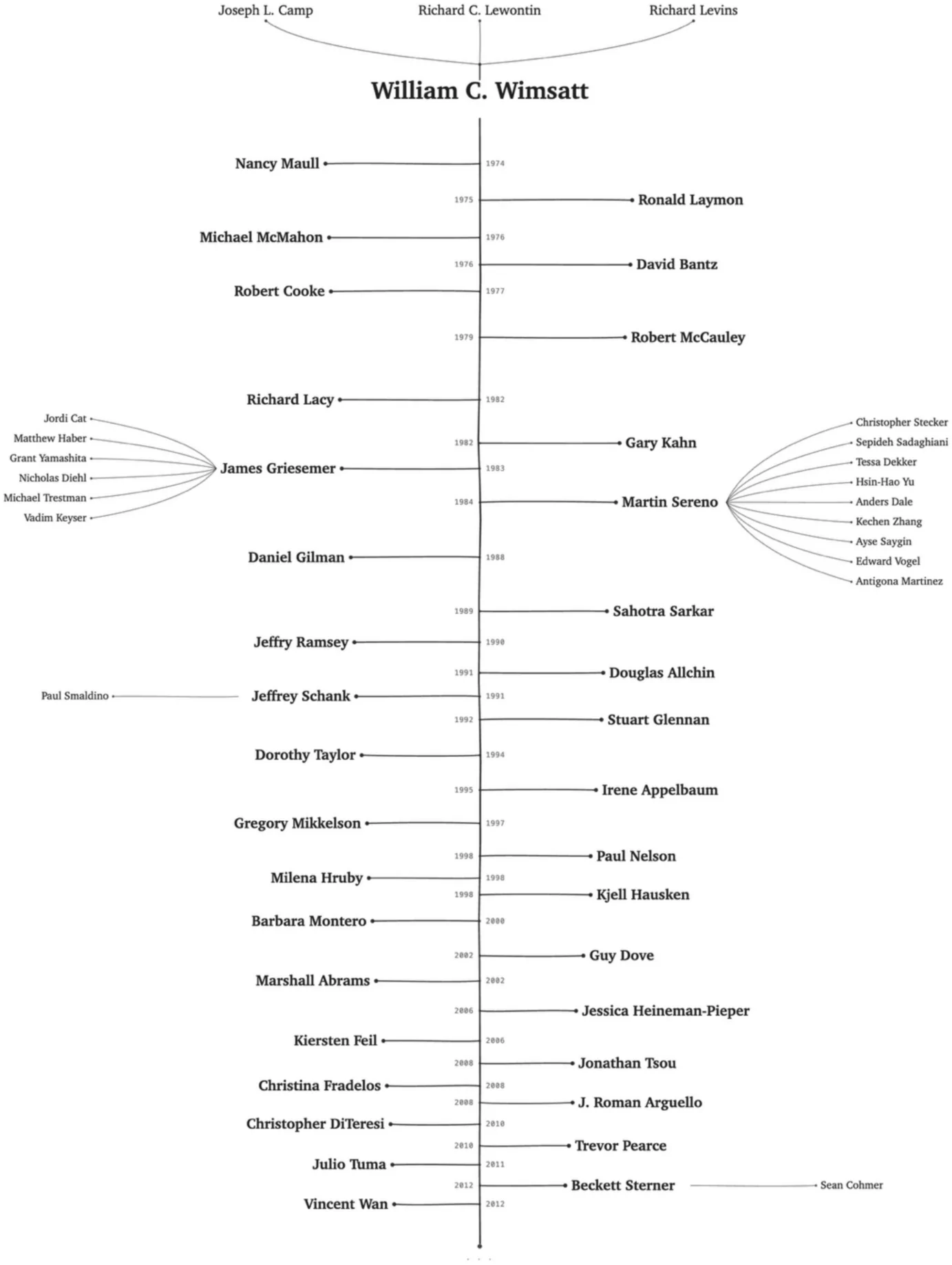
Scholars who have been scaffolded or are scaffolded by Bill Wimsatt

We hope our analysis may scaffold further investigations in education and in domains other than jazz and science. One question worth exploring is how the persistent need for social scaffolding should shape educational institutions. Another is whether our model transfers to other domains of expertise, high in the dimensions of sociality and improvisation, such as foreign-language acquisition and team sports. Although our argument focuses on only two case studies: jazz and science, we believe that our arguments extend at least to Bill Wimsatt’s research and the tradition of work in philosophy of biology he helped facilitate. His relationships with his students, as well as the numerous philosophy-of-biology interest groups are central parts of the structural scaffolds for their developmental trajectories and are still scaffolding their current research (see Fig. 4 for his formal students). On higher levels, the Committee on Conceptual Foundations of Science at the University of Chicago and the communities of philosophers of biology Bill has curated are still institutional scaffolds for our scholarship today.

## Data Availability

No datasets were generated or analysed during the current study.

## Footnotes

1 Thanks to a kind reviewer, who suggested that use of the language of “scaffolds” typically carries implications of impermanence. However, we wish to emphasize that there is continuity in the structures, institutions, and practices used to build and that sustain expertise; thus, our use of this term is intended to illuminate this continuity.

2 One reviewer notes that we seem to use the terms “skill,” “expertise,” and “competence” interchangeably. We grant that we are not as precise here as we should be (arguably, skills and mere competence are only necessary but not sufficient for expertise), but for ease of exposition, we will be using these terms in the “generic” sense. That is, sometimes one is referred to as “skilled” when they have achieved expert development of various skilled capacities.

3 Psychologists grant that the concept of scaffolding has evolved, such that psychologists use it in a variety of ways. However, in most introductions, surveys, or overviews of the use of the term we have been able to find, psychologists typically refer to scaffolding as “temporary.” See, e.g., Van de Pol, J., Volman, M., & Beishuizen, J. (2010). Scaffolding in teacher–student interaction: A decade of research. *Educational psychology review*, *22*(3), 271–296.: “Borrowed from the field of construction, where a scaffold is a temporary structure erected to help with the building or modification of another structure, the use of scaffolding as a metaphor within the domain of learning refers to the temporary support provided for the completion of a task that learners otherwise might not be able to complete. This support can be provided in a variety of manners that for example includes modeling and the posing of questions for different subjects (e.g., science, social studies) at different ages.”

4 Though Bickhard does not define these terms as precisely as we would like, “functional scaffolding” is a process in which “selection pressures” (e.g., environmental conditions, challenges) are “blocked” (e.g., removed) to allow for the learner to arrive at “constructions” (i.e., skilled solutions) to simpler problems, that can form a basis for more complex skill development. In Bickhard’s words: “Functional scaffolding… is the blocking of selection pressures in the service of making the (recursive) construction of competence or knowledge easier, or, perhaps, possible.” (2005, 170). “Self-scaffolding” is the capacity to carry out by oneself one or more of the steps typically described as depending on another in the scaffolding literature. Thus, for instance, Bickhard writes, “ self-scaffolding is perfectly possible if simple cases, idealizations, moving to subproblems, and so on are recognized as forms of scaffolding. An individual may well be able to do these sorts of scaffolding for him- or her-self.” (2005, p. 170).

5 The relation between SC and training activity could be either etiological or constitutive, corresponding, respectively, to Varga (2025, chapter 5)’s “Active Scaffolding (Manipulability)” and “Active Scaffolding (Mutual Manipulability),” which Varga introduces to allow for the possibility that a scaffold is sometimes best understood as a distinct cause, and sometimes, as an “extended” part of the cognition of the learner. We take active scaffolding in both senses to be distinct from a scaffolding trajectory.

6 This application of the concept of scaffolding episodes is relative to the psychologists’ interest of understanding children’s understanding of the symbolic meaning of gesture. The boundary of the episode may vary when the interest shifts. Psychologists interested in motor learning or personality may identify different sets of SC, training activities, and competence in this story.

7 Ericsson defines deliberate practice to contrast it with mindless repetition. Mindless repetition will often lead to incorrect postures, inefficient movements, and misplaced attention. One must consciously seek to listen to the elements previously less conscious of and move the body in ways that are less intuitive at first. Students also need evaluative mechanisms that help them understand whether they are doing it correctly.

8 The scaffolding in this case consists first of internal or external representations of the ideal result. At first, recognition and movement are slow because students must rely on intermediate symbols, tools, or heuristics for correct recognition or motor performance. However, over time, this becomes ingrained and becomes part of muscle memory.
